# Expanding the use of Twitter for medical education

**DOI:** 10.3402/meo.v21.33010

**Published:** 2016-08-24

**Authors:** Irene Moraitis, Miriam I. Zegeye

**Affiliations:** 1 Faulty of Medicine, Imperial College London, Sir Alexander Fleming Building, South Kensington Campus, London, SW7 2AZ, United Kingdom; 2 Faulty of Medicine, Imperial College London, London, United Kingdom

We have read Galiatsatos et al.'s recent article (1) about the use of Twitter and other social media with great interest, as they have the power to greatly enhance medical education. It is encouraging to see the large uptake of the Twitter page by the residents and the continued use of it.

Twitter, as a universal resource, allows the quick and easy dissemination of information worldwide. It has the capability to help create a global medical community where high-class institutions can share their resources freely with less advantaged medical communities.

We have already witnessed medical education attempt to keep up with the advances in the digital sector with the introduction of eLectures, ePortfolios and so on; however, the full potential of social media has yet to be tapped. This scope spans far wider than the residents Galiatsatos studied and could include undergraduate studies. Twitter could allow medical students to stay up to date with relevant advances regardless of which placement or hospital they are in. This is particularly relevant to the UK where students rotate through a number of different hospitals within the same medical school. Furthermore, the fact that Twitter posts are limited to a ‘bite-sized’ 140 characters can be used to make research more accessible and applicable to the students’ clinical experiences. The use of Twitter could be further expanded to include useful teaching aids such as mnemonics. This has already been used by a number of Instagram users with over 251,000 followers (@doctordconline), which shows great potential for other social media platforms.

With the vast majority of students having reported using social media (2), Twitter would enable the easy advertisement of educational events and research opportunities as well as being able to directly ask questions on a transparent platform for all who are following to see the response.

As with any technology, there are obstacles that need to be overcome. The most important one with regard to Twitter is confidentiality. For example, if a post refers to case-based teaching, patients may still be identifiable by their disease or demographics, especially in smaller hospitals. The lack of censorship may also pose a threat to the institution's reputation. Both these issues have already been noted with Facebook (3).

There is, of course, scope to take the use of social media even further in the healthcare community. As public health and health promotion are currently trending, the power of Twitter and other social media can be harnessed by national departments of health to reach the population. Moreover, to prevent the aforementioned issues, a social media platform for sole use by healthcare professionals could be developed.

*Irene Moraitis* Faulty of Medicine Imperial College London Sir Alexander Fleming Building South Kensington Campus London, SW7 2AZ United Kingdom Email: Irene.moraitis10@imperial.ac.uk *Miriam I. Zegeye* Faulty of Medicine Imperial College London London United Kingdom
